# Identification of Two Variants c.2697A > C and c.3305A > C in USP7 by Analysis of Whole-Exome Sequencing in Chinese Patients with Hao-Fountain Syndrome

**DOI:** 10.1055/s-0043-1778089

**Published:** 2024-01-16

**Authors:** Mei Sun, Qing Li, Ying Zhang, Yingzi Cai, Yan Dong, Jianbo Shu, Dong Li, Chunquan Cai

**Affiliations:** 1 Graduate College of Tianjin Medical University, Tianjin Medical University, Tianjin, Peoples' Republic of China; 2 Tianjin Children's Hospital (Children's Hospital of Tianjin University), Tianjin, Peoples' Republic of China; 3 Department of Neurology, Tianjin Children's Hospital (Tianjin University Children's Hospital), Tianjin, Peoples' Republic of China; 4 Academy of Medical Engineering and Translational Medicine, Tianjin University, Tianjin, Peoples' Republic of China; 5 Tianjin Pediatric Research Institute, Tianjin Children's Hospital (Tianjin University Children's Hospital), Tianjin, Peoples' Republic of China; 6 Tianjin Key Laboratory of Birth Defects for Prevention and Treatment, Tianjin Children Hosptial, Tianjin, Peoples' Republic of China

**Keywords:** *USP7*, development delay, intellectual disability, whole-exome sequencing, Hao-Fountain syndrome

## Abstract

**Background** Variants of ubiquitin-specific protease 7 ( *USP7* ) gene in humans are associated with a neurodevelopmental disorder—Hao-Fountain syndrome, its core symptoms including developmental delay, intellectual disability, and speech delay. Other variable symptoms can affect multiple systems. In present study, we report two patients with core features from two unrelated consanguineous families originating from the Tianjin Children's Hospital.

**Methods and Results** Genomic DNA was extracted from the peripheral blood samples collected from the probands with their family members and whole-exome sequencing (WES) was used to detect the pathogenic genes in the probands. Suspected variants were subsequently validated by Sanger sequencing. In family 1, WES revealed that the proband carried the de novo variant c.2697A > C (p.Leu899Phe) in *USP7* (NM_003470.3). In family 2, WES identified the variant c.3305A > C (p.Asn1102Thr) in *USP7* (NM_003470.3) from the proband.

**Conclusion** We reported two cases of Hao-Fountain syndrome caused by novel *USP7* variants. In addition, we report the first case of mosaicism with a *USP7* variant in Chinese family. Our findings demonstrate the importance of WES in diagnosis of genetic diseases and expands the *USP7* variants spectrum in Hao-Fountain syndrome. Moreover, we summarize the cases caused by *USP7* variants in the literature. Our study can provide a vital reference for the diagnosis of future cases.

## Introduction

The *USP7* gene is located on chromosome 16p13.2, it contains 31 exons and encodes a protein called ubiquitin-specific protease 7 (USP7) or herpesvirus-associated ubiquitin-specific protease 7. 1 The USP7 protein, weighing 135 kDa and consisting of 1,102 amino acids, belongs to the bi-ubiquitinase family. It serves as a crucial component of the MAGE-L2-TRIM27 ubiquitin ligase complex and possesses the ability to cleave multiple connections within ubiquitin chains. Furthermore, it plays an essential role in WASH-mediated intracellular actin assembly and protein cycle. 2 Previously, USP7 has been shown to regulate ubiquitination of proteins, which is important for DNA repair, transcription, and cancer. 3 4 The dysregulation of ubiquitin–proteasome system is implicated in the pathogenesis of numerous human diseases, including cancer and neurodegenerative diseases, and viral diseases. 5 USP7 plays a key role in crucial intracellular processes, including epigenetic regulation, cell cycle control, cellular growth and survival. It exhibits the ability to stabilize diverse tumor suppressor factors, and its upregulation is associated with numerous carcinogenic events. According to this feature, USP7 has become a potential target for cancer therapeutics. 6 7 8 9 10 Besides, disruption of *USP7* in humans is associated with a neurodevelopmental disorder.

Hao-Fountain syndrome is a neurodevelopmental disorder. The common phenotypes of this disease include developmental delay, intellectual disability, speech delay, mild facial deformity, and abnormal behavior such as autism. Other variable features contain decreased muscle tension, eating problems, walking slow and gait instability, male hypogonadism and eye disease, such as strabismus. Some patients had seizures, and some patients had mild white matter abnormalities in brain imaging. 11

In this paper, we reported two variants c.2697A > C and c.3305A > C in USP7 by WES analysis in Chinese patients with Hao-Fountain syndrome. We described the clinical characteristics of two patients and summarized their phenotypes, and also, we improved our understanding of the relationship between genotypes and phenotypes of clinical *USP7.*

## Materials and Methods

### Editorial Policies and Ethical Considerations

This study was approved by the Ethics Committee of Tianjin Children's Hospital. Written informed consent to participate in this study and for publication were obtained from the parents of the patients. The study complied with Chinese bioethics laws as well as the Helsinki declaration and its later amendments.

### Whole-Exome Sequencing and Sanger Sequencing

Five members were recruited in Tianjin Children's Hospital. Genomic DNA was extracted from the peripheral blood using a Blood Genomic DNA Mini kit (CoWin Biosciences, Jiangsu, China) according to the manufacturer's protocol. WES and Sanger sequencing was commercially supported by Guangzhou KingMed company and Mygenostics company. The pathogenicity classification of the screened gene variants was identified according to the criteria for the American College of Medical Genetics (ACMG) guidelines. The functional effect prediction and changes in 3D model molecular structure of protein caused by variants were obtained by PolyPhen-2 web server and Pymol.

## Results

### Case Presentation

**Fig. 1:**
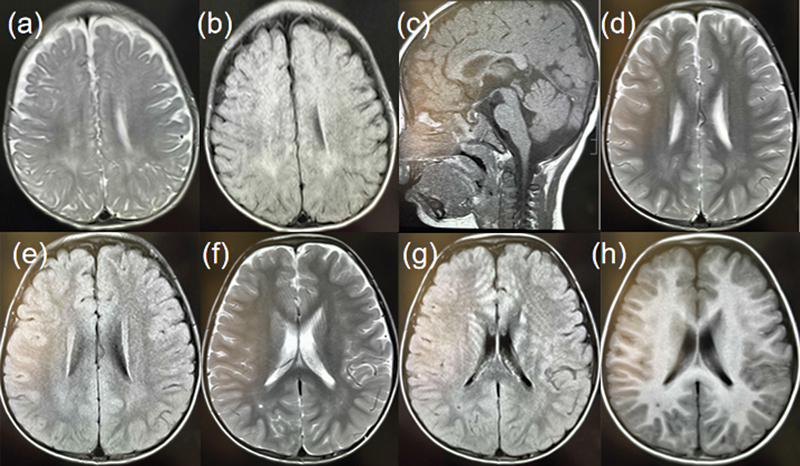
Clinical features. ( **a–c** ) The brain MRI of P1 showed delayed development of myelin sheath, widened extraventricular space, and patchy signal in pituitary. ( **d–h** ) The brain MRI of P2 showed the widening of perivascular space, the slight widening of extracerebral space, and the thickening of bilateral maxillary sinus, ethmoid sinus, and sphenoid sinus mucosa.

**Family 1:** The patient one (P1) was a 1-year-old boy; he was the only child born to healthy non-consanguineous parents after a late pregnancy with a history of intrauterine hypoxia. He has normal body weight and body length at birth. However, after 9 months of age, he gradually showed symptoms of developmental delay, displaying less activity compared with the children of the same age. Currently, at 1 year and 3 days old, he was unable to stand independently or perform fine motor tasks such as pinching objects between his thumb and forefinger or waving goodbye. Physical examination showed his body weight was 9 kg and head circumference was 44 cm. Gesell development scale evaluation for children aged 0 to 6 years suggested that he had a borderline state of developmental delay. Imaging examination demonstrated that the patient's heart had congenital patent foramen ovale and double superior vena cava. MRI showed delayed development of myelin sheath, widened extraventricular space, and patchy signal in pituitary ( Fig. 1a–c ). In addition, he also had left eustachian tube inflammation and red papules on the skin.

**Family 2:** The patient two (P2) was a 4-year-old boy born without asphyxia after full-term pregnancy. At 2 years, his parents found that his psychomotor movement lagged behind other children, especially the language development. Until the age of 3, he only could say two-character reduplicated words. He was reluctant to communicate with others and behaved frightened when facing strangers. He had been treated in a local hospital for language training. Now he is 4 years and 9 months old, the previous situation has not improved. Currently, he is unable to go up and down the stairs alone or control urination, whether awake or asleep. Twenty days before admission, his parents found that he had tremor and he walked and fell occasionally. The muscle tension of his limbs decreased and was evaluated as level IV. The brain MRI displayed the widening of perivascular space, the slight widening of extracerebral space, and the thickening of bilateral maxillary sinus, ethmoid sinus, and sphenoid sinus mucosa ( Fig. 1d–h ). Gesell development scale evaluation prompted he had mild developmental delay. The Autism Scale indicated the boy had mild to moderate symptoms of autism. Family history revealed that the patient's uncle and grandmother had similar symptoms of autism. Besides, his right ear hearing was slightly decreased with otitis media.

### Genetic Analysis

**Fig. 2:**
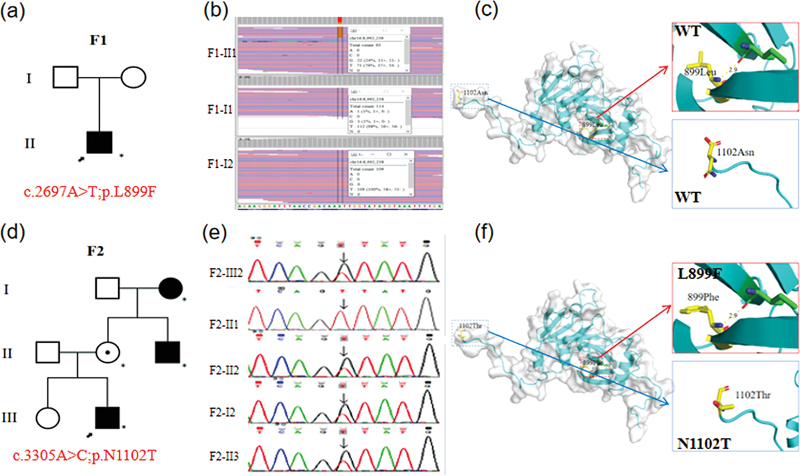
Molecular findings. ( **a** ) The pedigree chart in family 1. ( **b** ) WES detected a variant c.2697A > C in the proband (F1-II1). ( **c** ) 3D modeling revealed the wild type of USP7. ( **d** ) The pedigree chart in family 1. The proband (F2-III2), his uncle (F2-II3) and grandmother (F2-I2) carried the variant c.3305A > C and had autism symptoms. The mother (F2-II2) carried the same variant but was healthy. ( **e** ) Sanger sequencing in family 2. The result showing the c.3305A > C variants in USP7 identified in proband (F2-III2) and his mother (F2-II2), uncle (F2-II3) and grandmother (F2-I2). ( **f** ) 3D modeling revealed the variants L899F and N1102T of USP7. WES, whole-exome sequencing.

We performed WES on the two families, the variant c.3305A > C was validated by Sanger sequencing using the DNA of the members in family 2 ( Fig. 2b, e ), the pedigree chart in two families was shown ( Fig. 2a, d ). Protein Data Bank (PDB) structure revealed the wild type and two novel variants Leu899Phe and Asn1102Thr in USP7 ( Fig. 2c, f ). In family 1, the child is a mosaic with a mutation rate of 23.7%. The results disclosed a de novo missense variant c.2697A > C in the exon 25 of *USP7* gene in P1, leading to the amino acid substitution p.(Leu899Phe). Besides, it was absent from healthy controls in the 1000 Genomes Project Database, ESP6500siv2_ALL database, ExAC_ALL database, gnomAD_genome_ALL database, or dbSNP147 database. Bioinformatics prediction results tend to be harmful. The c.2697A > C variant is classified as uncertain (PM2_Supporting, PS2_supporting, PP2, BP4) according to the ACMG standard guidelines.

In P2, we detected a heterozygous variant in c.3305A > C in the exon31 of *USP7* , leading to the amino acid substitution p.(Asn1102Thr). The result revealed that the variant was inherited from his mother. Furthermore, the variant was also discovered in grandmother and uncle. The ClinVar database had no pathogenic analysis results of the site. Bioinformatics prediction results tend to be harmful. The pathogenicity of the variant was evaluated as uncertain by the ACMG standard guidelines (PM2_Supporting, PP1, PP2, PP3).

### Summary of Published and Present Variants of *USP7*

By reviewing previous reports and comparing them with our patients, 11 12 13 14 15 we summarized the main clinical features and signs of Hao-Fountain syndrome in Table, including developmental delay/intellectual disability (30/31, 96.8%), speech delay (30/30, 100%), dysmorphic face (20/22, 84.6%), brain MRI anomalies (18/23, 78.3%), hypotonia (19/27, 70.8%), behavioral anomalies (19/27, 70.4%), eye abnormalities such as esotropia, myopia, strabismus, nystagmus (16/27, 59.3%), autism spectrum disorder (11/20, 55.0%), gastroesophageal reflux with feeding difficulties (11/20, 55.0% and 13/25, 52.0%, respectively). Abnormal behavior consisted of aggressive behavior, temper tantrums, impulsivity, compulsivity, stubbornness, manipulative behavior. 11 In Zheng's research, 15 abnormal behavior manifested as impaired social interactions and poor eye contact. Other symptoms such as abnormal gait (7/17, 41.2%), difficulty weight (10/22, 45.5%) are also common. Some patients also have epilepsy (11/30, 36.7%), attention deficit hyperactivity disorder (7/17, 36.8%), chronic constipation (6/19, 31.6%), and neonatal poor suck (6/21, 28.6%). In the reported literature, Zampieri et al reported a female patient with isolated tubal torsion. 14 In Zheng et al's article, 15 two male patients had hydrocele testis and chordee with hypospadias, respectively, all included in the Table of hypogonadism as statistics. Symptoms of respiratory system contains sleep apnea/sleep disturbance (6/23, 26.1%), asthma (6/17, 35.3%). Nearly one quarter of patients had small hands (5/21, 23.8%), small feet (5/22, 22.7%), short stature (6/23, 26.1%), and scoliosis or kyphosis (7/23, 30.4%). A low proportion of variable symptoms only occurred in individual patients, which may be accidental.

## Discussion

At the beginning, our diagnosis was not smooth. Due to the rarity of the disease and the atypical symptoms, we were initially unable to identify the correct cause of the two patients. WES revealed a missense variant c.2697A > C p.(Leu899Phe), which further affects the coding product and destroys the protein function. It is likely to be an important genetic basis for the symptoms of developmental delay. P1 was a mosaicism with a mutation rate of approximately 23.7%. The clinical manifestations of P1 included mild developmental delay and white matter abnormalities in brain imaging, no obvious other symptoms were related to Hao-Fountain syndrome. In theory, the higher the proportion of abnormal cell mosaicism, the more severe the symptoms. On the one hand, the child is too young to show many symptoms, his age may not reach the onset period of many disease. On the other hand, it was considered to be related to the low proportion of mosaicism, thus showing mild symptoms. 16 Mosaicism is defined by the existence of more than one cell population in a single individual causing genetic or genomic changes in a single zygote, which is a common biological phenomenon. 17 Reduced penetrance in mosaic primary immunodeficiencies was first recorded in a patient with delayed-onset adenosine deaminase deficiency in the 1980s and 1990s. 18 19 With the accumulation of different patients, the mosaic phenomenon was strengthened as evidence of the mechanism of decreased penetrance. 20 We speculate the reason that fewer symptoms of P1 are influenced by mosaicism. Although no studies have confirmed, the mosaicism of pathogenic alleles to reduce the clinical penetrance cannot be ignored. In family 2, the WES results revealed that P2 and his mother and uncle and grandmother carried the variant c.3305A > C, but not all individuals were affected. The grandmother and uncle have autism symptoms similar to P2 while the mother is healthy and has no phenotype, which is inconsistent with the autosomal dominant inheritance. The mother did not meet the conditions of age before onset and subclinical phenomena. Therefore, the lack of penetrance can explain why the mother carried the *USP7* variant but no symptoms. Besides, autism patients may show emotional abnormalities, negative emotions even contribute to the development of heart disease. Therefore, psychotherapy is as important as physical therapy. 21

According to the related symptoms of Fountain's statistics, 11 speech delay, general developmental delay, and abnormal behaviors are the characteristics of the disease, and the rest are variable symptoms, in addition to the symptoms listed in the Table. In our patients, the P1 had patent foramen ovale and double superior vena cava. In Zheng et al's paper, patent foramen ovale was also found in a patient; 15 similar congenital cardiac structural abnormalities have also been reported in individual patients, including mitral valve prolapse, patent ductus arteriosus, atrial septal defect, and ventriculomegaly. Besides, P1 had hearing loss in the right ear, which was the same as the previous report 12 13 ( Table 1 ).

**Table 1:** Summary of the clinical characteristics of patients with pathogenic *USP7* variants

*USP7* variants	Deletion ( *n* = 8)	Truncating ( *n* = 7)	Missense ( *n* = 10)	Splice site ( *n* = 4)	Our patients Missense ( *n* = 2)	Total ( *n* = 31)
Gender	5M, 3F	2M, 5F	4M, 6F	4F	2M	13M,18F
**Development**						
Speech delay	8/8	7/7	10/10	4/4	2/2	30/30
DD/ID	8/8	7/7	9/10	4/4	2/2	30/31
Nonverbal	1/8	0/4	4/9	0/3	0/2	4/24
Decreased fetal movement	0/6	2/4	1/3	0/3	0/2	3/18
Neonatal hypotonia	1/6	6/6	4/7	0/3	0/2	11/25
Hypotonia	4/7	6/6	5/7	1/3	1/2	17/24
**Behavior**						
Behavioral anomalies	7/8	3/5	3/9	2/3	1/2	19/27
Autism spectrum disorder	7/7	0/4	2/4	0/3	1/2	11/20
Skin picking	2/8	0/5	1/4	0/3	0/2	3/23
ADHD	3/7	1/3	1/5	2/3	0/2	7/19
**Neurologic**						
Brain MRI anomalies	3/4	4/6	9/9	0/2	2/2	18/23
Seizures	4/8	3/7	3/9	1/4	0/2	11/30
Abnormal gait	2/5	1/3	4/4	0/3	0/2	7/17
**Craniofacial**						
Dysmorphic facial features	4/6	6/6	7/9	4/4	0/2	22/26
Eye anomalies	6/8	5/6	4/8	1/3	0/2	16/27
Hearing difficulties	1/7	2/5	0/8	0/3	0/2	3/25
**Gastrointestinal**						
Feeding problems	4/7	3/5	4/8	2/3	0/2	13/25
Difficulty gaining weight	1/6	2/4	5/7	2/3	0/2	10/22
Neonatal poor suck	2/6	3/6	1/4	0/3	0/2	6/21
Chronic constipation	3/5	2/3	1/6	0/3	0/2	6/19
GERD	3/5	3/4	4/6	1/3	0/2	11/20
Excessive weight gain	0/6	0/5	0/6	3/3	0/2	3/22
Chronic diarrhea	0/4	0/2	2/5	0/3	0/2	2/16
**Skeletal**						
Contractures	2/6	3/5	0/4	0/3	0/2	5/20
Small hands	2/6	3/5	0/5	0/3	0/2	5/21
Small feet	1/6	3/5	1/6	0/3	0/2	5/22
Short stature	2/7	1/5	1/7	0/3	0/2	6/23
Scoliosis or kyphosis	2/6	1/5	3/6	3/3	0/2	7/23
Hip dysplasia	0/6	1/5	2/8	0/3	0/2	3/24
**Respiratory**						
Sleep apnea/sleep disturbance	3/7	1/4	1/7	0/3	0/2	6/23
Asthma	1/4	2/4	1/4	2/3	0/2	6/17
**Urinogenital**						
Hypogonadism (including cryptorchidism/micropenis)	5/7	1/3	3/6	1/4	0/2	10/22

Abbreviations: ADHD, attention-deficit hyperactivity disorder; DD/ID, developmental delay/intellectual disability; F, female; GERD, gastroesophageal reflux disease; M, male; MRI, magnetic resonance imaging; *USP7* , ubiquitin-specific protease 7.

In short, we reported two variants c.2697A > C and c.3305A > C of USP7 and broadened the variant spectrum. Mosaicism and the decreased penetrance of diseases caused by unknown reasons will increase the difficulty of diagnosis of rare genetic diseases—it will lead to missed diagnosis and misdiagnosis of diseases. In clinical practice, WES can provide reliable evidence for the precise diagnosis of diseases and genetic counseling.

## References

[1] Hao Y H Fountain M D Jr Fon Tacer K USP7 acts as a molecular rheostat to promote WASH-dependent endosomal protein recycling and is mutated in a human neurodevelopmental disorder Mol Cell 2015 59 06 956 969 26365382 10.1016/j.molcel.2015.07.033 PMC4575888

[2] Hao Y H Doyle J M Ramanathan S Regulation of WASH-dependent actin polymerization and protein trafficking by ubiquitination Cell 2013 152 05 1051 1064 23452853 10.1016/j.cell.2013.01.051 PMC3640276

[3] Li M Chen D Shiloh A Deubiquitination of p53 by HAUSP is an important pathway for p53 stabilization Nature 2002 416 6881 648 653 11923872 10.1038/nature737

[4] Nicholson B Suresh Kumar K G The multifaceted roles of USP7: new therapeutic opportunities Cell Biochem Biophys 2011 60(1-2):61 68 21468693 10.1007/s12013-011-9185-5

[5] Colland F Formstecher E Jacq X Small-molecule inhibitor of USP7/HAUSP ubiquitin protease stabilizes and activates p53 in cells Mol Cancer Ther 2009 8 08 2286 2295 19671755 10.1158/1535-7163.MCT-09-0097

[6] Harakandi C Nininahazwe L Xu H Recent advances on the intervention sites targeting USP7-MDM2-p53 in cancer therapy Bioorg Chem 2021 116 105273 34474304 10.1016/j.bioorg.2021.105273

[7] Hernández-Pérez S Cabrera E Salido E Correction: DUB3 and USP7 de-ubiquitinating enzymes control replication inhibitor Geminin: molecular characterization and associations with breast cancer Oncogene 2019 38 24 4886 31068665 10.1038/s41388-019-0753-2

[8] Nicklas S Hillje A L Okawa S A complex of the ubiquitin ligase TRIM32 and the deubiquitinase USP7 balances the level of c-Myc ubiquitination and thereby determines neural stem cell fate specification Cell Death Differ 2019 26 04 728 740 29899379 10.1038/s41418-018-0144-1 PMC6460386

[9] Song M S Salmena L Carracedo A The deubiquitinylation and localization of PTEN are regulated by a HAUSP-PML network Nature 2008 455 7214 813 817 18716620 10.1038/nature07290 PMC3398484

[10] Xia X Liao Y Huang C Deubiquitination and stabilization of estrogen receptor α by ubiquitin-specific protease 7 promotes breast tumorigenesis Cancer Lett 2019 465 118 128 31518603 10.1016/j.canlet.2019.09.003

[11] Fountain M D Oleson D S Rech M E Pathogenic variants in USP7 cause a neurodevelopmental disorder with speech delays, altered behavior, and neurologic anomalies Genet Med 2019 21 08 1797 1807 30679821 10.1038/s41436-019-0433-1 PMC6752677

[12] Capra A P Agolini E La Rosa M A Novelli A Briuglia S Correspondence on “Pathogenic variants in USP7 cause a neurodevelopmental disorder with speech delays, altered behavior, and neurologic anomalies” by Fountain et al Genet Med 2021 23 02 421 422 33012787 10.1038/s41436-020-00978-x

[13] Priolo M Mancini C Pizzi S Complex presentation of Hao-Fountain syndrome solved by exome sequencing highlighting co-occurring genomic variants Genes (Basel) 2022 13 05 889 35627274 10.3390/genes13050889 PMC9141324

[14] Zampieri N Pulvirenti R Pedrazzoli E Camoglio F S Hao-Fountain syndrome and genital disorders: report of a new possible association Ital J Pediatr 2022 48 01 182 36273155 10.1186/s13052-022-01367-7 PMC9588229

[15] Zheng H Mei S Li F Expansion of the mutation spectrum and phenotype of *USP7* -related neurodevelopmental disorder Front Mol Neurosci 2022 15 970649 36466803 10.3389/fnmol.2022.970649 PMC9708884

[16] Rio M Royer G Gobin S Monozygotic twins discordant for submicroscopic chromosomal anomalies in 2p25.3 region detected by array CGH Clin Genet 2013 84 01 31 36 23061379 10.1111/cge.12036

[17] Liu A Yang X Yang X Mosaicism and incomplete penetrance of *PCDH19* mutations J Med Genet 2019 56 02 81 88 30287595 10.1136/jmedgenet-2017-105235 PMC6581080

[18] Arredondo-Vega F X Kurtzberg J Chaffee S Paradoxical expression of adenosine deaminase in T cells cultured from a patient with adenosine deaminase deficiency and combine immunodeficiency J Clin Invest 1990 86 02 444 452 1974554 10.1172/JCI114730 PMC296746

[19] Uberti J Peterson W D Jr Lightbody J J Johnson R M A phenotypically normal revertant of an adenosine deaminase-deficient lymphoblast cell line J Immunol 1983 130 06 2866 2870 6854019

[20] Gruber C Bogunovic D Incomplete penetrance in primary immunodeficiency: a skeleton in the closet Hum Genet 2020 139(6-7):745 757 32067110 10.1007/s00439-020-02131-9 PMC7275875

[21] Karki M Mahara G Heart diseases, anxiety disorders, and negative thoughts Heart Mind (Mumbai) 2022 6 01 22 25

